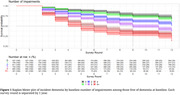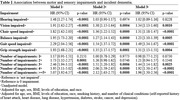# Longitudinal Associations between Sensory Difficulty and Motor Impairment and Incident Dementia in the National Health and Aging Trends Study

**DOI:** 10.1002/alz.087654

**Published:** 2025-01-09

**Authors:** Anis Davoudi, Ryan J. Dougherty, Amal A Wanigatunga, Yuri Agrawal, Hang Wang, Nicholas S Reed, Joshua R Ehrlich, Alden L. Gross, Jennifer A Schrack

**Affiliations:** ^1^ Johns Hopkins Bloomberg School of Public Health, Baltimore, MD USA; ^2^ Center on Aging and Health, Johns Hopkins University, Baltimore, MD USA; ^3^ University of Colorado School of Medicine, Aurora, CO USA; ^4^ University of Michigan, Ann Arbor, MI USA

## Abstract

**Background:**

Impairments in sensory and motor function are common and have been independently linked with higher risk of dementia in older adults. Yet, there is limited information associated with the increasing number of such impairments and dementia risk. This study investigated longitudinal associations between sensory and motor impairment and dementia in older adults.

**Method:**

We used data from the US National Health and Aging Trends Study (NHATS), a nationally representative cohort study of community‐living Medicare beneficiaries aged 65 years and older. Baseline sensory impairments were defined based on self‐reported vision and hearing loss indicators. Baseline motor impairments were defined based on performance in gait, repeated chair stands, standing balance, and grip strength tests. Multivariable Cox regression models were used to evaluate the hazard ratio of incident dementia from baseline (2011) to 2022.

**Result:**

A total of 3847 participants without dementia at baseline were included (weighted mean age 73.9 (95% CI: 73.7‐74.1) years old and 56% (95% CI: 54.1‐57.9) female). Compared to those without impairment in each specific domain, hazard ratios for incident dementia were 1.02 (95% CI 0.89‐1.16, p = 0.82) for hearing impairment, 1.34 (95% CI 1.13‐1.60, p = 0.001) for vision impairment, 1.31 (95% CI 1.18‐1.47, p<0.001) for chair stand impairment, 1.23 (95% CI 1.10‐1.39, p<0.001) for standing balance impairment, 1.49 (95% CI 1.34‐1.67, p<0.001) for gait speed impairment, and 1.31 (95% CI 1.13‐1.51, p<0.001) for grip strength impairment (Table 1). Additionally, compared to participants with no impairments, the hazard ratio for incident dementia for those with three impairments was 1.44 (95% CI 1.14‐1.82, p = 0.003), with four impairments was 1.92 (95% CI 1.50‐2.47, p<0.001) and with 5‐6 impairments was 1.96 (95% CI 1.50‐2.56, p<0.001) (Table 1, Figure 1).

**Conclusion:**

In this cohort study of US Medicare beneficiaries, vision and motor impairment as well as a higher number of impairments were associated with elevated risk of dementia. These findings underscore the cumulative burden of sensory and motor impairments on incident dementia. A better understanding of the temporality and longitudinal effect of sensory and motor impairments on incident dementia may help inform future targeted interventions.